# Potential for the development of light therapies in mild traumatic brain injury

**DOI:** 10.2217/cnc-2018-0006

**Published:** 2018-10-15

**Authors:** Adam C Raikes, William DS Killgore

**Affiliations:** 1 Social, Cognitive & Affective Neuroscience Lab, Department of Psychiatry, College of Medicine, University of Arizona, Tucson, AZ, USA; 2 ORCID: 0000-0002-1609-6727; 3 ORCID: 0000-0002-5328-0208

**Keywords:** blue light, brain injury, circadian rhythm, concussion, fatigue, mild TBI, phototherapy, sleep–wake disruption

## Abstract

Light affects almost all aspects of human physiological functioning, including circadian rhythms, sleep–wake regulation, alertness, cognition and mood. We review the existing relevant literature on the effects of various wavelengths of light on these major domains, particularly as they pertain to recovery from mild traumatic brain injuries. Evidence suggests that light, particularly in the blue wavelengths, has powerful alerting, cognitive and circadian phase shifting properties that could be useful for treatment. Other wavelengths, such as red and green may also have important effects that, if targeted appropriately, might also be useful for facilitating recovery. Despite the known effects of light, more research is needed. We recommend a personalized medicine approach to the use of light therapy as an adjunctive treatment for patients recovering from mild traumatic brain injury.

Mild traumatic brain injuries (mTBIs) are currently among the most socially, medically and academically talked-about issues today. The annual mTBI incidence is at least 1.5 million reported injuries in the USA [1–3]. However, this number fails to capture the untold number of such injuries that likely go unreported [4,5]. The long-term consequences – plausibly including neurodegenerative conditions [6–10], impaired cognitive abilities [11–13] and altered psychosocial functioning [14–20] – necessitate the need for efficacious treatments following injury and proactive preventative methods for reducing injury risk and consequence.

Despite significant job-, school- and economic-related burdens associated with the medical management of mTBIs [1], there is no currently accepted gold standard treatment for mTBIs [21,22]. Historically, the treatment of choice was total rest to allow the brain to heal. However, recent research advances are giving way to more active treatments, with an emphasis on early intervention [23–25]. While early reports are promising for short-term management, the long-term impact of these active interventions is not well established. Additionally, there is little information on methods of optimizing these active approaches or complementary treatments that may enhance recovery in both the short and long term.

One complementary treatment method involves the use of light exposure. Light, both visible and invisible, can have powerful effects on numerous neurological and physiological systems [26–29]. Additionally, light is potentially a modifiable aspect of the environment in which one exists to allow for optimal healing, recovery and a return to homeostatic states following mTBI. The purpose of this narrative review is to provide a focused overview of the role and effect of light on neurological processes and to connect these effects with potential areas of intervention with respect to mTBI.

## The fate of ambient light: image-forming & nonimage-forming pathways

**Figure 1.**
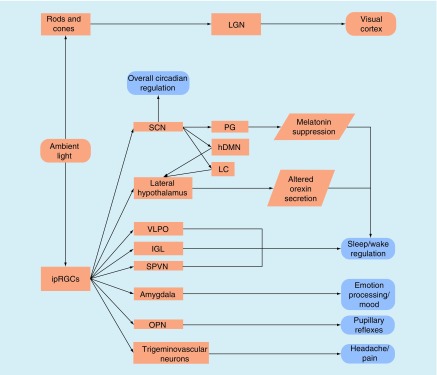
**Schematic representation of the destinations for ambient light entering the eye.** hDMN: Hypothalamic dorsomedial nucleus; IGL: Intergeniculate leaflet; ipRGC: Intrinsically photosensitive retinal ganglion cell; LC: Locus coeruleus; LGN: Lateral geniculate nucleus; OPN: Olivary pretectal nucleus; PG: Pineal gland; SCN: Suprachiasmatic nucleus; SPVN: Supraparaventricular nucleus; VLPO: Ventrolateral preoptic nucleus.

The primary sensory function of the eyes is to translate information contained in light into images [30]. This is accomplished primarily through two classes of retinal photoreceptors. Cones are color-sensitive photoreceptors while rods respond to changes in brightness and are particularly sensitive to dim light. Light information is converted by these photoreceptors, resulting in the stimulation of retinal ganglion cells (RGCs) that project to subcortical nuclei, including the lateral geniculate nucleus of the thalamus, ultimately terminating in the primary visual cortex and visual attention networks (Figure 1). These pathways provide the sensory information for vision and terminate in areas that process and interpret those sensory signals. For a complete review of the transformation from light information to visual interpretation, please see reference [30].

An additional class of photoreceptor was discovered in the early 2000s and is the starting point for the nonimage-forming (NIF) pathway [31–34]. This third type of photoreceptor is expressed directly by a small proportion of RGCs (termed intrinsically photosensitive RGCs; ipRGCs). These ipRGCs are maximally sensitive to blue light (λ = 460–480 nm) and less so to longer wavelengths including green, amber and red [31]. ipRGCs, combined with information regarding illuminance and color from the rods and cones, then directly project to regions involved in the regulation of or influence the actions of (Figure 1):

- Circadian rhythms. Projections from the ipRGCs to the suprachiasmatic nucleus (SCN), the primary biological clock for all circadian processes, can directly induce entrainment of either expected or aberrant circadian rhythms [31,35].
- Melatonin suppression. Stimulation of ipRGCs results in melatonin suppression via the SCN’s projections to the pineal gland as well as the paraventricular nucleus of hypothalamus and superior cervical ganglion [34,36–38].
- Sleep–wake cycle regulation. In conjunction with the above-mentioned melatonin suppression pathways, direct projections from the ipRGCs go to the ventrolateral preoptic nucleus, subparaventricular nucleus and lateral hypothalamus [34]. The SCN may additionally influence the action of the hypothalamus’s dorsomedial nucleus, and locus coeruleus, affecting the lateral hypothalamus secretion of orexin [33,34].
- Cognition. The aforementioned pathways regulating circadian rhythms, melatonin suppression and sleep–wake cycles additionally exert both direct and indirect influences on cognition and alertness [26].
- Emotional processing and mood. The amygdala, a primary site of emotional processing and integration, receives direct ipRGC projection [34,39].
- Intracranial nociception. ipRGCs project to the trigeminovascular neurons of the thalamus that transmit nociceptive information from the dura to the cortex [40].
- Pupillary constriction. ipRGCs directly project to the olivary pretectal nucleus [32–34], which in turn project to the Edinger–Westphal nucleus. The cumulative action of this pathway is pupillary constriction. For a complete review of the effects of mTBI on the pupillary light reflex, including NIF pathway contributions, please see [41].

As can be seen, light has the powerful potential to alter numerous biological and cognitive processes through this NIF pathway. Given the complex interactions between circadian timekeepers, hormone and neurotransmitter secretion pathways, cognition, and emotions, light has the potential to positively or negatively influence how individuals function at a very basic level. Consequently, using light as a therapeutic intervention has the potential to directly influence recovery and function following mTBI. In the following sections, we review potential areas of intervention and, where possible, expected outcomes from using light as a therapy.

## mTBIs & their consequences

mTBIs are a change or disruption in the normal functioning of the brain subsequent to an external force applied to the head or body [42,43]. Typical guidelines for distinguishing mTBIs from more severe TBIs include a mechanism indicative of mTBI; loss of consciousness <30 min (if at all); post-traumatic amnesia <24 h; Glasgow Coma Scale scores 13–15; and lack of gross abnormalities on traditional neuroimaging [42,44,45]. The effects of a single mTBI are often viewed as transient and may include somatic symptoms, sleep–wake disturbances, and cognitive and behavioral disruptions. However, while these effects are common, the individual manifestations of these are highly individualized and may depend on premorbid functioning and the location and mechanism of injury [46]. Additionally, many individuals experience persistent symptoms associated with an mTBI, and recent findings indicate that the incidence of long-lasting mTBI-related functional decrements may be underestimated [42]. Here we provide an overview of mTBI-related consequences that may be positively affected by light therapy, with an emphasis on sleep and sleep-related consequences given the previously identified NIF-pathway effects.

### Sleepiness & fatigue

High-quality sleep is an essential component of all aspects of human performance. Current recommendations for adequate sleep recommend 7–9 h of sleep per night for adults. Despite these recommendations, chronic sleep loss (<5.5 h/night of sleep) in the USA is reaching epidemic levels [47]. For individuals with chronic sleep loss, the consequences are numerous including increased somatization [48,49], poor emotional processing and responsiveness (e.g., increased incidence of depression and anxiety) [50–52], impaired cognition (vigilant attention, executive function, working and long-term memory) [53–56] and poor motor performance [57–59], as well as increased risk for general health issues including diabetes [60–62], cardiovascular disease [60,63], neurodegeneration [64,65] and overall poorer quality of life [66]. While the exact nature of this trend toward chronically undersleeping is not fully understood, work–life stress (e.g., increased expectations for high job-related hours, social stress) as well as the highly prevalent use of fluorescent lighting and blue-shifted light-emitting diode screens at night [67–69] are all implicated.

Compounding the endemic social issue of chronic sleep loss, detrimentally altered sleep is among the most common short- and long-term consequences of mTBI [70–74]. Indeed subjectively perceived traumatic brain injury (TBI)-related sleep–wake disruption is reported by plausibly as many as 70% of all individuals who sustain a TBI (regardless of severity) [70,74,75]. Individuals with mTBI commonly self report insomnia [74–79] and hypersomnolence (excessive sleepiness) [71,75,80–86], though hypersomnia [80,85,87,88] and circadian rhythm sleep disorders [82,89] are also reported. Objectively, these reports are often corroborated by poor sleep efficiency, higher than usual wake after sleep onset and sleep latency, as well as more fragmented sleep and changes in sleep architecture [87,90–95]. Clinically, it is important to recognize that post-mTBI insomnia may be misdiagnosed as a circadian phase issue, specifically delayed sleep phase syndrome [89]. Consequently, individuals sustaining an mTBI may be at an increased risk for all of the aforementioned sleep-related health and performance outcomes without treatment for mTBI-related alterations.

The mechanisms by which mTBI induces altered sleep are not fully understood. However, there are implications from both human studies and animal models that suggest any combination of possible mechanisms including altered circadian hormone regulation (e.g., melatonin release) [96–98] and reductions in neurotransmitter function (e.g., loss of or damage to wake-promoting, orexin-secreting neurons in the hypothalamus) [99–101] among others may be responsible.

In addition to these possible mechanisms of post-mTBI sleep changes, sleep loss or low-quality sleep may impede and impair healing following mTBI. There is considerable evidence indicating that decreases in sleep quantity and quality, both in humans and animals as well as apart from and in relation to mTBI, may impair hippocampal neurogenesis, disrupt ATP production thereby extending the mTBI-initiated neurometabolic cascade [102,103], prolong neuroinflammation [104], impede metabolic waste removal in the brain [105], alter cerebrovascular responsiveness and compromise glymphatic removal of phosphorylated tau [105–107]. Collectively, these effects of sleep disruption may contribute to the short- and long-term clinical presentation of mTBI as well as precipitate the neurodegenerative conditions, particularly tau-related pathologies (e.g., chronic traumatic encephalopathy), commonly thought to be associated with repetitive head trauma.

### Alertness

As noted, an mTBI may induce a sequela whereby disrupted circadian rhythms lead to sleep dysfunction, culminating in daytime sleepiness or fatigue. Broadly, daytime fatigue is associated with decreased alertness and vigilant attention capabilities. Indeed, a recent study demonstrates that evidence of increased fatigue and decreased alertness in an mTBI sample are closely related concepts that are difficult to disentangle [86]. Furthermore, mTBI is associated with degraded alertness and vigilance in both the short and long term [86,108,109]. With regards to daytime alertness, phototherapy may provide a nonpharmacological route for improving daytime functioning in post-mTBI individuals.

### Cognition

mTBIs additionally exert a substantial, negative impact on various cognitive functions, including working memory, attention, executive function and visuospatial processing [20,110–117]. While deficits in these cognitive domains are generally resolved soon after injury (e.g., most within a month, many within 3 months postinjury), there is evidence to suggest subtle, persistent deficits that linger well beyond this clinically accepted time course. Additionally, there are individuals in whom the full impact of these deficits does not resolve quickly.

Apart from mTBI, increasing sleep need as well as sleep deprivation conditions induce marked deterioration in the cognitive capabilities of individuals [53,118–120]. Given the impact of mTBI on sleep, daytime sleepiness and fatigue, it is reasonable to posit that many of the observed cognitive deficits, particularly those that linger beyond the general clinical time course, may be mediated by sleep-related changes.

### Depression

Depression and increased reporting of depressive symptoms are common following mTBI. The incidence of post-mTBI depression may be as high as 42% in adults and 22% in children and adolescents [121–124]. Premorbid depression is a risk factor for prolonged recovery from mTBI and may be associated with postconcussion symptoms, as well as sleep disruption, impaired cognition and other post-mTBI psychiatric symptoms (e.g., anxiety) [17,20,125–129]. Therefore, ameliorating post-mTBI depression may improve overall symptom presentation and be associated with improvements in sleep and cognitive function.

### Post-traumatic headache & pain

Post-traumatic headaches (PTH) and chronic pain are among the most common symptoms experienced by individuals recovering from mTBI. The incidence of PTH likely may be as high 90% [130–135], and the incidence of chronic pain may be as high as 75% [136]. Additionally, both PTH and chronic pain may mediate, or be mediated by, post-mTBI poor sleep, daytime sleepiness, cognitive deficits and depression [122,137–142]. Consequently, the aforementioned benefits of bright or blue light therapy for sleep, cognitive performance and depression may have positive effects on PTH and pain.

## The effects of different types of light & applications to mTBI

Given the range of deficits and changes observed following mTBIs, as well as the known NIF pathways for light, light therapy has the potential to positively influence a wide range of cognitive, emotional and physiological functions. Below, we discuss the known effects of various aspects (colors, intensities) of light across the range of human performance. These findings are additionally summarized in Table 1.

### Polychromatic white light

Polychromatic white light is essentially a broad-spectrum light. Because white light includes nearly all wavelengths, it also encompasses the blue light portion of the spectrum (∼460–448 nm) that selectively activates ipRGCs and therefore has important circadian and hormone-secreting properties [31]. Consequently, it could be expected that white or bright light therapy would induce changes in post-mTBI circadian rhythms, sleep and alertness.

A recent meta-analysis indicates that light therapy in general is effective in the treatment of sleep disorders that include circadian rhythm sleep disorders, insomnia, and sleep problems associated with Alzheimer’s disease and dementia [27]. This meta-analysis included randomized controlled trials and within-subject design studies utilizing polychromatic white light, and blue-enriched white light, as well as several studies utilizing monochromic blue light. Overall, positive effects for light therapy were observed for circadian shifts, bed and wake times, sleep onset latency, total sleep time, wake after sleep onset, sleep efficiency, sleepiness and alertness, sleep quality, insomnia symptoms and fatigue [27]. The authors additionally report that light intensity (ranging from 2000 to 10,000 lux in the majority of included studies) had positive effects on individuals with insomnia, with greater intensity increasing the beneficial effects of light therapy [27]. In estimating effect sizes, the authors did not distinguish the effects of bright light from those of blue light; however, only 9% of the included studies specifically examined blue light.

An additional systematic review by Souman *et al.* examined the effects of light therapy on alertness. Across the reviewed literature, there is the indication that increasing the intensity of polychromatic white light significantly increases subjective alertness [28]. However, this does not appear to translate into improved vigilance or reaction. Additionally, there is limited evidence for significant improvements in subjective or performance-based measures of alertness with blue-shifted polychromatic white light [28]. Cumulatively, these findings suggest that the intensity of polychromatic white light may have positive effects on subjective alertness.

Furthermore, polychromatic white light therapy has been shown to be effective in reducing depressive symptoms in individuals with diagnosed depression, including major depressive disorder. Recent meta-analyses indicate that light therapy, especially polychromatic white light, reduces depressive symptoms at post treatment compared with control participants and this effect is more pronounced for standalone light therapy than for studies using light as an adjunctive therapy [29,143]. The effect is additionally stronger when light therapy is used in the morning than any other time [29]. These effects were observed over studies including polychromatic white, green and pale blue light [29,143].

### Monochromatic blue & blue-shifted white light

As previously noted with polychromatic white light, blue light therapy – including monochromatic blue and blue-enriched white light – alters circadian rhythms, particularly the timing of melatonin release, in individuals with a variety of sleep-disrupted conditions [27,144]. These studies demonstrate that short amounts (30 min or more) of focused and intentional daily light therapy in the morning effectively advances individuals’ circadian rhythms, evidenced by the timing of melatonin secretion. In general, the effects of phototherapy are condition dependent, but may include changes in circadian rhythm and improvements in sleep duration, self-reported sleep quality, insomnia symptoms and fatigue [27].

In addition to the direct effects of monochromatic blue light therapy on sleep quantity and quality, the appropriate timing of melatonin secretion is essential for maintaining normal daytime arousal and minimizing fatigue. There is robust evidence that blue-wavelength light is effective in acutely decreasing sleepiness and fatigue [145–152] as well as increasing concentrations of arousal-promoting hormones (e.g., cortisol) [153]. Furthermore, blue light therapy has positive effects on increasing alertness [145,147,149,152,154–169]. This phenomenon is present both during the day (e.g., morning blue light exposure) and night.

Prior work has additionally demonstrated that blue light therapy increases activation in cognition-related, task-specific brain regions [157,159,170–176]. However, while light affects brain activation, the actual behavioral effects of blue light exposure are not quite as clear. Individual studies have demonstrated improvements in cognitive performance on working memory, digit recall, sustained attention and arithmetic tasks while others have shown no improvements or even reduced performance in response to light exposure [145,158,169,176–184]. It is thus unclear the extent to which blue light therapy may directly affect cognitive performance beyond those conferred by improvements or alterations in sleep, fatigue or overall alertness.

With respect to mood and affect, blue or blue-shifted light can variously cause [143,185] or improve [29,186] depressive symptoms depending on the timing of therapy (e.g., when timing coincides with or in opposition to naturally expected patterns). Mood disorders, including depression, are associated with the homeostatic maintenance of circulating stress hormones. Among these, glucocorticoids like cortisol exhibit circadian rhythmicity, with a night-time accumulation period and clearance during the day [187,188]. Thus, blue light that influences circadian rhythms, as previously described for sleep and melatonin, may impart a beneficial effect on glucocorticoid expression when utilized in circadian-optimal timings or may induce or worsen mood disorders when mistimed (e.g., night-time use of light-emitting diode screens).

However, one potential pitfall in the application of blue light therapy as described to this point is the exacerbation of PTH. The blue light-sensitive ipRGCs directly project to the trigeminovascular neurons in the thalamus [40]. Prior research related to migraine indicates that these neurons transmit nociceptive signals originating in the dura to cortex, thereby contributing to the perception of intracranial pain during a migraine [40]. Furthermore ipRGC inputs onto the trigeminovascular neurons may modulate the response to light by migraineurs. This neural mechanism may explain why individuals feel worse when exposed to light and preferentially seek dark rooms for relief (photophobia) when experiencing a migraine. While the overarching neural mechanisms of mTBI-related PTH resemble, but may not be exactly the same as those for migraine [140], light-based exacerbation of PTH and/or photophobic responses by individuals post-mTBI may likely have the same neural underpinning. Thus it is plausible that, despite the numerous potential benefits of blue light on circadian rhythms, fatigue, alertness and cognition following mTBI, blue-light or blue-shifted white light treatments may be poorly tolerated and may indeed worsen PTH in some individuals. At present, this specific possibility has not been directly explored in treatment studies using blue light for treating symptoms of mTBI, but research on this topic would be a welcome addition to the literature.

### Monochromatic red light

For individuals seeking to enhance alertness without modifying their circadian rhythm (e.g., increasing daytime alertness in the presence of a normal circadian rhythm), utilizing blue light therapy may have unintended and unwanted effects, primarily on melatonin secretion. Interestingly, prior work has shown that longer wavelength light (e.g., red light) may have equally powerful alerting effects [157,160–164]. Red light is detected by L-cones in the retina, and the ipRGCs that are sensitive to blue light are not sensitive to the longer wavelengths (∼630 nm) of red light [31,33]. In some preliminary work, the alerting effects of red light were present both in the late afternoon and at night, and were comparable to the effects of blue light. While the mechanisms by which red light has an alerting effect are not fully understood, a plausible explanation is that it may influence the actions of subcortical regions apart from SCN resulting in alerting effects unrelated to melatonin secretion or suppression [159,170].

### Monochromatic green light

While the use of blue light may exert its most profound effects on circadian phase advancement or resetting circadian rhythms that mediate sleep, blue light specifically suppresses melatonin secretion thereby inhibiting or delaying the actual onset of sleep. Though possibly beneficial for altering post-mTBI sleep timing or reducing daytime sleepiness and fatigue, this effect on melatonin does nothing for actually promoting night-time sleep. On the other hand, preliminary evidence from animal models suggests that green light (∼530 nm) indeed has a sleep-promoting function [189]. This has not yet been confirmed in human studies and the specific mechanisms are not described as yet, though multisynaptic M-cone projections to the ventrolateral preoptic area (involved in sleep promotion) and lateral hypothalamus (where wake-promoting orexin is secreted) may plausibly create this relationship [189,190].

As previously noted, blue light may also have the unintended consequence of aggravating PTHs. However, further research with migraineurs demonstrates that the use of green light has a positive effect on migraine symptoms, including at a minimum no exacerbation of the headache and at best a decrease in the intensity of symptoms [191]. This effect is observed relative to the use of white, blue, amber and red light. Additionally, animal models have demonstrated that green light confers antinociceptive benefits, both at the sensory threshold and with neuropathic pain [192]. Therefore, individuals with mTBI-related PTH or pain may benefit either from environments bathed in green light or from glasses, which preferentially filter the spectra of incoming light to preferentially include green light.

## Light therapy following mTBI

To date, two published studies have specifically examined the effects of light therapy following mTBI. Sinclair *et al.* exposed participants to 45 min of morning blue or yellow light for 4 weeks [193]. They demonstrated that individuals receiving blue light, as opposed to yellow light or no treatment, reported less daytime fatigue and sleepiness, faster response times on a sustained psychomotor vigilance task, less self-reported sleep disruption and lower self reports of depression symptoms at 2 and 4 weeks than at baseline. These findings suggest that, for post-mTBI individuals who do self-report fatigue, daytime sleepiness or sleep disruption, daily blue light therapy may be an effective nonpharmacological method for improving function in these areas. It is unclear, however, whether these effects persist after treatment cessation.

Additionally, a study by Bajaj *et al*. had mTBI participants use blue-wavelength light therapy or an amber-wavelength placebo light for 30 min every morning for 6 weeks and found that it was associated with significant changes in white matter integrity (as measured by water diffusion along axonal tracts) within the corpus collosum, corona radiata and thalamus [194]. Moreover, for those receiving blue-light treatment, the magnitude of white matter changes was associated with greater sleep latency on the multiple sleep latency test, which is an objective measure of biological sleepiness. Additionally, they found that the increases in white matter integrity were associated with an improvement in delayed memory performance, but only among those receiving the blue light treatment. These associations were not significant among those receiving the amber light placebo condition. In other words, 6 weeks of blue light therapy appeared to increase the integrity of axonal white matter, and this change was associated with decreased tendency to fall asleep during the day as well as improved delayed memory performance.

Furthermore there is some evidence suggesting that there may be positive effects of blue light exposure on anxiety following mTBI [195,196]. In fact, even a single 30-min exposure to blue-wavelength light appears to increase activation of the anterior cingulate cortex when anticipating positive stimuli compared with an amber placebo light, which might help explain the mood and anxiety improvements that follow blue light exposure [196]. Thus, limited evidence suggests that blue-wavelength light may be effective for some aspects of recovery from mTBI, but more research is needed before the benefits of this approach are fully understood.

Extrapolating the findings from both healthy individuals and those with other neurological conditions as well as animal studies, there may be additional unidentified benefits of light therapy for mTBI beyond those that have been specifically identified. Both polychromatic white and blue lights may be useful for resetting aberrant circadian rhythms, improving sleep, decreasing daytime sleepiness and fatigue, increasing alertness, and decreasing depressive symptoms. Red light may be beneficial for improving alertness without inducing circadian shifts, but more work is needed. Green light may help to promote night-time sleep, minimize PTH and reduce pain. However, given the paucity of studies on post-mTBI light therapy, these applications are speculative at best. Though there is the indication of positive effects on both neural and behavioral outcomes, these findings require further corroboration with larger studies and diverse mTBI populations. Future research objectives are presented in Box 1.

## Additional uses of light

In addition to the potential benefits of using visible light as a treatment method, low-level laser therapy (LLLT) has noted wound healing and anti-inflammatory properties, particularly in the near-infrared spectral range. While LLLT is beyond the scope of the present discussion, we encourage readers to see [197] for a recent review on the use of LLLT for TBI.

## Current limitations to using phototherapy for mTBI

As has been indicated in the preceding sections, there are numerous potential benefits to using light as an adjunctive therapy in the management of mTBIs. However, there are some limitations that currently limit the scale and scope of the inference that can be made regarding the effects of phototherapy in the recovery from mTBI. Notably, there is significant heterogeneity in light characteristics, timing, duration and illuminance of light therapy between studies that may all influence the outcomes of these studies [26,27,29,198]. Thus considerably more work is required, particularly for mTBI-related applications, to identify the optimal parameters that maximize the benefit to the individual.

Additionally, there is evidence that polymorphisms in the *PER3* clock gene may additionally explain interindividual differences in the relationship between light exposure and cognition [175]. Consequently, future studies and clinical applications of bright or blue light therapy, particularly for improving cognitive performance, should take genetic variations into account.

Furthermore, many studies employ some form of light box to deliver the phototherapy. These boxes are portable and allow individuals to be treated at home, which is a tremendous benefit. However, an unavoidable drawback to at-home treatments of this nature is compliance and adherence. Additionally, these boxes have limited spatial effectiveness and require the user to be within a certain distance from the light source in order to be effective. Consequently, treatment may be challenging or even impossible in individuals who are unable to remain in front of the light box for the treatment duration. Alternative light presentation methods, such as goggle-mounted light systems are currently being tested, and may afford greater ambulation and flexibility of use. At present, these devices have only been tested with a limited range of wavelengths and will require further research to determine their effectiveness.

Finally, safety concerns are always critical in deciding whether to use a particular treatment, and light therapy is no different. Some safety concerns have been raised for blue light, in particular. There is some evidence to suggest that retinal damage is possible with prolonged exposure to short-wavelength light. Though the optimal wavelength to stimulate ipRGCs (∼480 nm) is considerably greater than violet and ultraviolet wavelengths where damage is more certain, it is a plausible concern [199]. However, there is no evidence in the published literature of such damage from the types of therapy described here [200]. Some companies who manufacture blue-wavelength light-box devices have had their products independently evaluated for blue-light retinal hazard and have reported the exposure hazard to be minimal at the distance, intensity and duration of light emitted for those devices. Another consideration is that the amount of blue-wavelength light emitted by most light-box devices is considerably less than that obtained by an equal duration of exposure to midday outdoor sunlight. Additionally, as noted with photophobia and PTH, many individuals do report headaches and other somatic symptoms with the use of light boxes [200]. Although there is no compelling evidence at this time that standard light therapy devices pose a significant optical hazard when used according to manufacturer’s instructions, further research into the long-term safety and side effects of light exposure treatment is warranted. As with any treatment, decisions to engage in light therapy should involve judicious evaluation of the benefits and potential risks involved.

## Conclusions

Light is all around us and is an ingrained natural part of our biological rhythms and daily functioning. It is essential for image-forming vision and our ability to interact with the environment. The NIF impacts of light on physiology make it a powerful tool as well as a potent inhibitor of function. While considerable in-roads are being made to understand how early and active intervention may improve the outcomes from mTBIs, the individual’s environment is an oft-ignored but important consideration. Light is a critical feature of our environment that has a powerful influence on our biology. There are numerous, interconnected systems that are impaired or altered by mTBIs and whose function can be influenced by exposure to light. Outcomes from two small-scale trials indicate that daily blue-wavelength light therapy may be effective for reducing daytime fatigue and improving sleep and efforts are underway to corroborate these findings. Further evidence from other populations indicates that other wavelengths may confer additional therapeutic benefits (e.g., green light for improving post-traumatic headache) and such findings require future research specific to mTBI.

## Future perspective

We believe that significant advances in the use of light therapy can and will be made in the decade ahead to leverage these advantages and minimize the drawbacks. It is our position, though, that in order for light therapies to be effective for mTBI, or any condition, they must be specifically tailored to the individual (i.e., personalized medicine) and account for the uncontrollable aspects of the environment (e.g., sunlight during commuting, work environments with limited capacity to be modified). Accordingly, this requires a complete and ongoing needs assessment of the individual as well as an understanding of those aspects of the environment that can be adapted or modified to meet these needs.

For example, technological control of circadian lighting will require smart lights that shift dominant or active wavelengths throughout the day to mimic lighting patterns that more closely reflect sunlight while indoors (e.g., more blue light in the morning that gives way to more amber wavelengths in the late afternoon). These types of lights do not require a light box, but instead would completely replace existing ambient lighting methods. This technology could be leveraged at the home, or potentially the workplace, to ensure that ambient light maximizes the circadian benefit for the individual, and indeed entrains appropriate rhythms given individual needs. Furthermore, many of these circadian lighting systems could be internet connected. Therefore, tuning of ambient colors in response to an individual’s daily needs could be accomplished through needs and symptom reporting via an internet-ready device (e.g., tablet, cellular phone).

**Figure 2.**
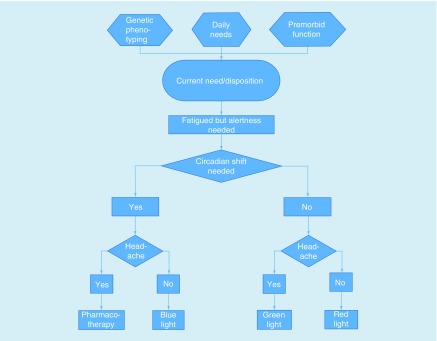
**Example decision tree for a precision medicine, needs-based approach to light therapy.**

Such a scenario would enable fine tuning the environment to meet daily, and even moment by moment, presentation of individualized mTBI-related symptoms. This may mean more blue in the morning for a person on a normal sleep–wake schedule, but for someone regularly engaged in shiftwork, this may mean more blue is presented in the evening as they prepare for work. Likewise, lighting could be manipulated to address symptom expression and behavioral needs, such as shifting to a more green-lit room if the individual reports pain or a headache on a given day or when falling asleep is a goal (Figure 2). Additionally, individuals could conceivably maximize cognitive performance and alertness by shifting to red light during the afternoon, evening or night for improved alertness without inducing an unwanted or unnecessary circadian shift that is associated with blue light.

In these scenarios, lighting patterns could be altered on a daily basis to reflect not only the individual’s unchangeable physiology (e.g., *PER3* phenotype, which may limit the effect of blue light on sleep and cognition) and overarching needs (e.g., necessary wake times), but also day-to-day changes in mTBI-related symptoms, fatigue and cognitive demands. In so doing, and in conjunction with other medical management, we can leverage the environment to maximize recovery following mTBI and return biological systems to homeostatic states rapidly to facilitate full returns to work, school, sport and life.

**Box 1.**  **Future research objectives in the development of light therapy for mild traumatic brain injury recovery.**

In light of the potential benefits of light therapy in mild traumatic brain injury recovery as well as the physiological nonimage-forming light pathways, the following research objectives are reasonable targets for exploration:

**Sleep-related research**

- Expanding current efforts to identify the effects of monochromatic blue light to correct aberrant circadian rhythms, improve sleep, reduce daytime fatigue and improve white matter integrity
- Identifying the optimal dosage and timing of blue light
- Identifying positive effects and differences between polychromatic white light and monochromatic blue light as pertains to circadian rhythms and sleep metrics
- Identifying the optimal dosage and timing for the use of green light to improve night-time sleep onset
- Identifying the optimal dosage and timing for the use of red light to improve alertness without circadian shifting

**Cognition**

- Identifying the optimal color, timing and dosage of light to improve short-term activation related to cognitive function

**Somatic**

- Identifying whether green light reduces the presentation of post-traumatic headache
- Identifying whether green light reduces comorbid pain

**Precision medicine**

- Identifying the personal characteristics that will lead to responsiveness to light treatment (e.g., *PER3* polymorphisms, degree of circadian dysrhythmia, level of daytime sleepiness/fatigue)
- Development and refinement of treatment parameters throughout the day (i.e., timing of blue vs red vs green light therapy based on current symptoms and needs)

**Executive summary**

**Mild traumatic brain injury, sleepiness & fatigue**

- Blue light is selectively absorbed by intrinsically photosensitive retinal ganglion cells. These cells project directly the suprachiasmatic nucleus and influence circadian rhythms and melatonin secretion.
- There is some evidence suggesting that mild traumatic brain injuries (mTBIs) induced circadian dysrhythmias.
- Targeted use of blue light can be used to shift circadian rhythms and improve nighttime sleeping and daytime fatigue.
- Emerging evidence suggests that green light may be an effective sleep promoter.

**mTBI & alertness**

- Blue light improves daytime fatigue and sleepiness, leading to greater alertness.
- Red light also improves alertness and may be useful when affecting circadian rhythms is undesired.

**mTBI & cognition**

- Blue light potentiates activation in areas associated with multidomain cognition. Further work is needed to more completely understand these mechanisms.

**mTBI & depression**

- Depression is a common post-mTBI complaint. Prior work demonstrates that blue light is effective in improving both seasonal and nonseasonal depression.

**mTBI, post-traumatic headache & pain**

- Despite the positive effects of blue light, the intrinsically photosensitive retinal ganglion cells also project to thalamic neurons that receive input from dural nociceptors that are active during migraine headaches. Blue light therapy may exacerbate post-traumatic headaches.
- Green light does not exacerbate migraine headaches and in some cases improves symptom presentation.

**Future perspective**

- Phototherapy has the potential to modify the brain’s functioning across a wide range of affected systems following mTBI.
- Technology exists that enables ambient lighting to be modified on demand to change the visible spectrum to meet needs.
- A personalized medicine approach – combining genetic phenotyping, injury characteristics, current symptoms and current needs to create an optimal ambient light profile – could make phototherapy a potent, ever-present aspect of mTBI management and recovery.
